# Supplementary material for: B-OK: A Visual and Tactile Tool for HIV Treatment Adherence Support in a United States Urban Center
Source: medRxiv. 2024 Mar 1:2024.02.28.24303498. Preprint. [Version 1] doi: 10.1101/2024.02.28.24303498 (PMC10925346; doi:10.1101/2024.02.28.24303498)

medRxiv preprint doi: https://doi.org/10.1101/2024.02.28.24303498; this version posted March 1, 2024. The copyright holder for this preprint (which was not certified by peer review) is the author/funder, who has granted medRxiv a license to display the preprint in perpetuity.
It is made available under a CC-BY 4.0 International license .

Supplementary Appendix

medRxiv preprint doi: https://doi.org/10.1101/2024.02.28.24303498; this version posted March 1, 2024. The copyright holder for this preprint (which was not certified by peer review) is the author/funder, who has granted medRxiv a license to display the preprint in perpetuity. It is made available under a CC-BY 4.0 International license .

Intervention Guide

Now I want to show you the B-OK bottles. Please feel free to look at or hold the bottles while I talk. These bottles are to help you understand what is happening inside your body.

Mix of black and red beads

- First, let's look at this bottle.
- It has a mix of black and red beads.
- The black beads show healthy cells in your body.
- The red beads show the HIV virus.
- This is what it looks like when most people first find out they have HIV, before starting treatment.
- They may feel completely normal, but the red beads show the virus is growing.
- If folks are having sex, they can pass HIV to their partners.

Mostly red beads

- If a person does not start HIV treatment, eventually their body will look like this red bottle.
- There are very few healthy black beads left.
- This might happen slow, or it might happen quickly, but it will happen eventually.
- At this stage, it is hard to fight off other sicknesses like cancer and infections.
- Folks often end up in the hospital and can even die.

Mostly black beads

- On the other hand, if a person starts HIV treatment, and takes their treatment every day, their body quickly becomes like this bottle.
- It is full of healthy black beads.
- This is what people mean when they talk about an "undetectable viral load" or a "suppressed viral load."
- You can see there is still one red bead, because there is not yet a cure for HIV.
- However, it is so small and deeply buried that it can't hurt you and it can't be passed on through sex, as long as you keep taking your HIV treatment.
- This is what people mean when they say "Undetectable=Untransmittable" or "U=U" — that if you are in this stage, you can't pass the virus through sex, even if you don't use a condom.

Supplementary Table 1. Changes in HIV-related knowledge, attitudes, intentions, and perceptions (with higher numbers indicating greater agreement to the questions) by whether participants were interviewed prior to exposure to B-OK, whether participants had optimal (>95%) self-reported adherence, and study site (community-based organization [CBO] vs university). Data are presented as mean (SD), with p-value testing whether changes were different by group (two-sample t-test, two-tailed).

	**Interviews**			**Self-Reported Adherence**			**Site**		
	Pre-Intervention Interview (N=20)	No Pre-Intervention Interview (N=98)	p-value	Optimal (N=73)	Sub-optimal (N=36)	p-value	Community-Based (N=55)	Clinic-Based (N=63)	p-value
**Knowledge**									
How well do you understand the term “viral suppression?”	2.1 (3.7)	2.5 (4.1)	0.68	2.5 (4.2)	2.3 (3.8)	0.82	2.1 (3.8)	2.7 (4.2)	0.40
How well do you understand the term “U=U” or “Undetectable=Untransmittable?”	0.7 (4.3)	1.6 (3.6)	0.34	1.5 (3.7)	1.3 (3.8)	0.71	1.2 (3.1)	1.6 (4.1)	0.57
Imagine a person living with HIV who is taking HIV treatment and has an undetectable viral load. How likely is it that this person would pass HIV to a regular sexual partner?	1.2 (4.3)	-0.3 (3.4)	0.09	-0.1 (3.9)	0.0 (3.0)	0.90	0.4 (4.4)	-0.4 (2.6)	0.21
Imagine a person living with HIV who is taking HIV treatment and has an undetectable viral load. How likely is it that this person would be harmed by HIV?	-0.2 (3.7)	-0.1 (3.9)	0.92	-0.2 (4.4)	-0.2 (3.1)	0.98	-0.4 (3.8)	0.1 (3.9)	0.46
**Attitudes**									
Agrees with: I worry that HIV medications do not completely eliminate the risk of getting HIV through sex	1.3 (4.6)	-1.2 (4.3)	0.02	-0.5 (4.8)	-1.3 (3.9)	0.34	-1.9 (4.7)	0.1 (4.0)	0.02
Agrees with: Having HIV means I will eventually die from it	-1.1 (4.3)	-0.8 (3.1)	0.70	-0.6 (3.5)	-1.2 (3.1)	0.40	-0.5 (2.6)	-1.2 (3.9)	0.26
**Intentions**									
Imagine you were taking HIV treatment and had an undetectable viral load. How likely would you be to use a condom to prevent passing HIV to a sex partner who did not have HIV?	-0.6 (3.7)	-1.3 (2.9)	0.36	-0.9 (3.0)	-1.6 (3.1)	0.28	-1.4 (3.1)	-0.9 (2.9)	0.39
Agrees with: I feel motivated to stay on (or restart) HIV treatment	0.4 (1.4)	0.1 (1.7)	0.44	0.0 (1.9)	0.4 (1.5)	0.31	0.1 (1.9)	0.1 (1.5)	0.96
**Perceptions**									
Agrees with: I understand my illness	0.2 (1.2)	0.2 (1.7)	0.95	-0.0 (1.4)	0.7 (2.0)	0.04	0.1 (2.0)	0.2 (1.1)	0.75

Supplementary Figure 1. Histogram showing pre- and post-intervention responses on a zero to ten scale to the question: “How well do you understand the term ‘viral suppression?’”

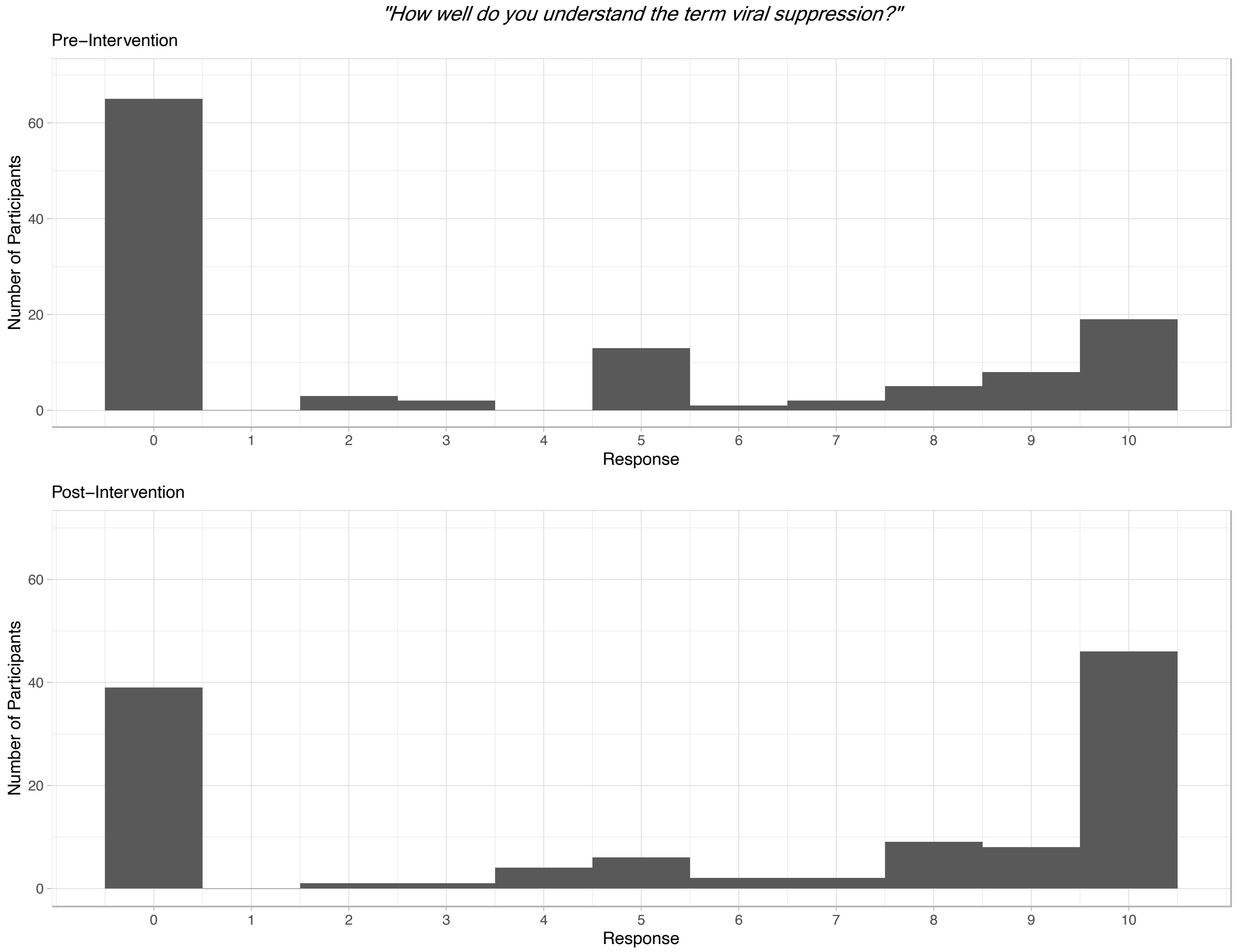

Supplementary Figure 2. Histogram showing pre- and post-intervention responses on a zero to ten scale to the question: “How well do you understand the term ‘U=U or Undetectable=Untransmittable?’”

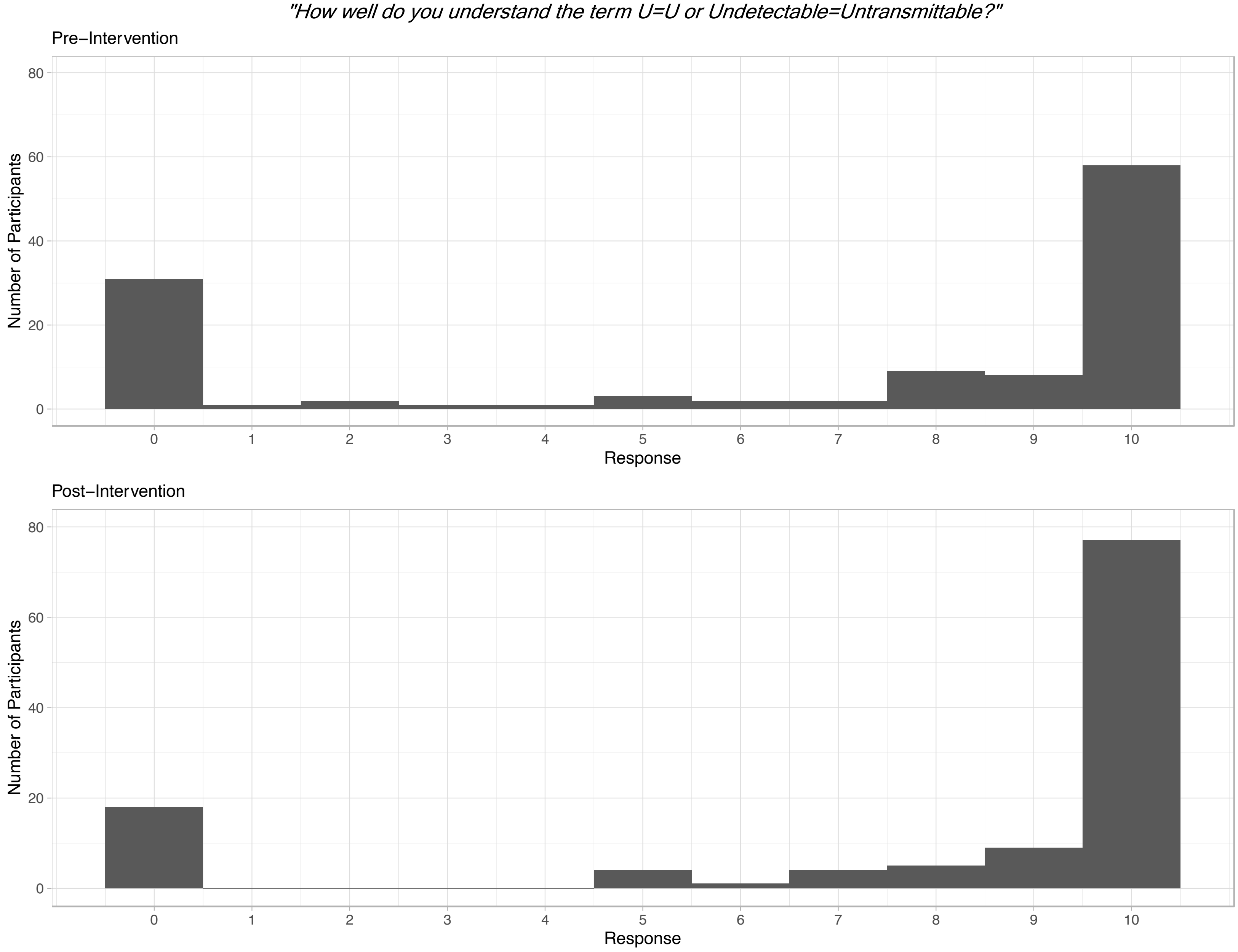

Supplementary Figure 3. Histogram showing pre- and post-intervention responses on a zero to ten scale to the question: “Imagine a person living with HIV who is taking HIV treatment and has an undetectable viral load. How likely is it that this person would pass HIV to a regular sexual partner?”

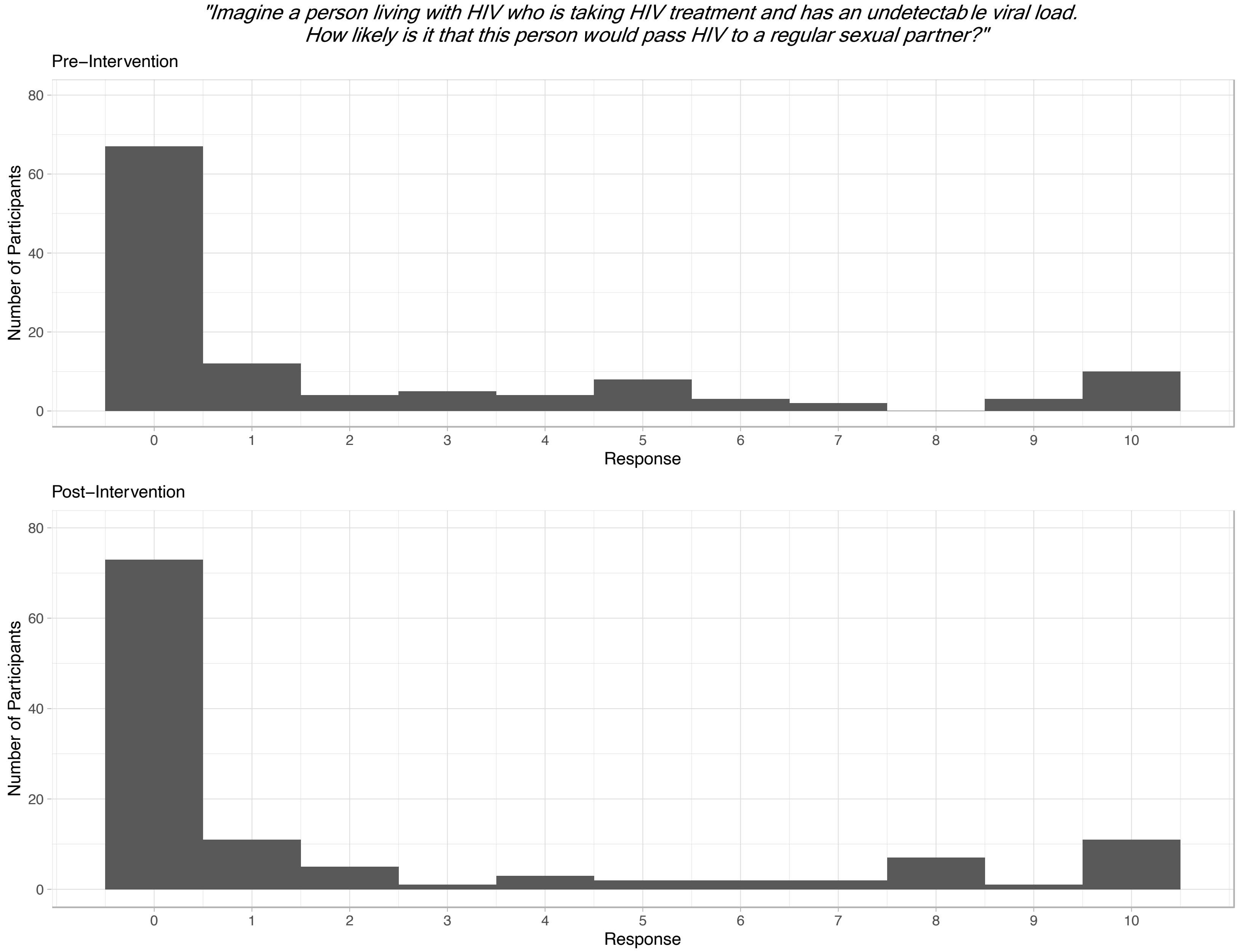

Supplementary Figure 4. Histogram showing pre- and post-intervention responses on a zero to ten scale to the question: "Imagine a person living with HIV who is taking HIV treatment and has an undetectable viral load. How likely is it that this person would be harmed by HIV?"

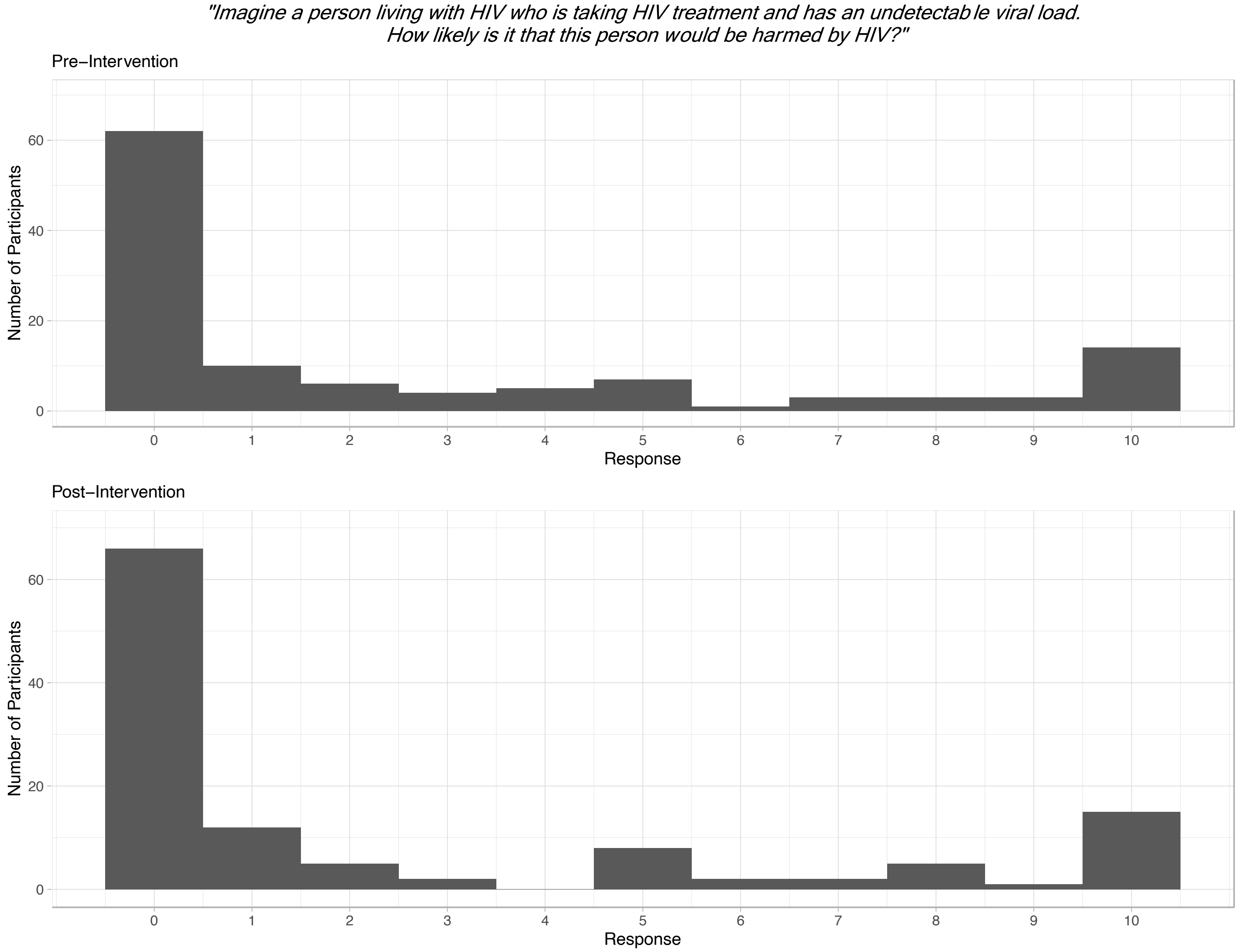

Supplementary Figure 5. Histogram showing pre- and post-intervention responses on a zero to ten scale to the question: "Agrees with: I worry that HIV medications do not completely eliminate the risk of getting HIV through sex."

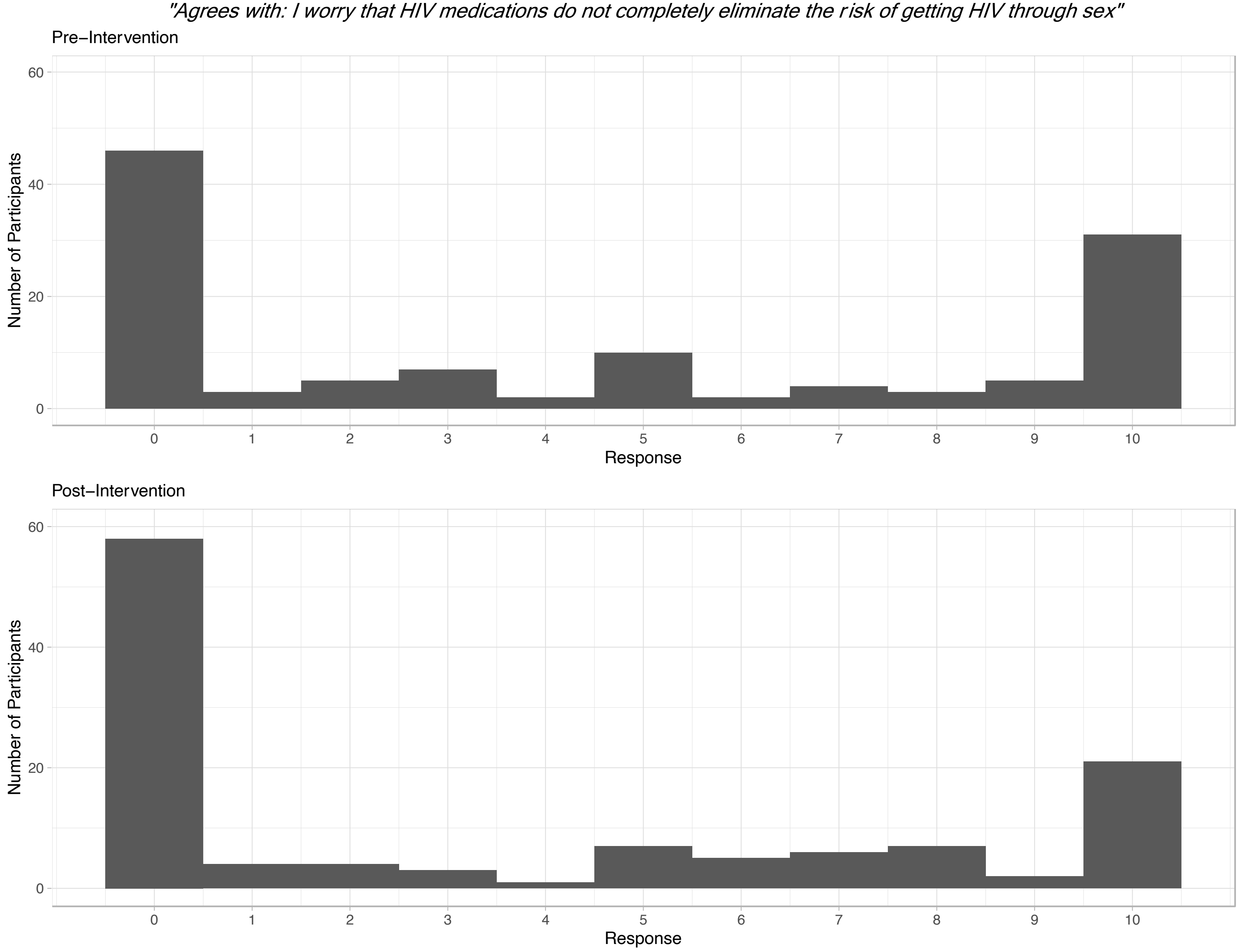

Supplementary Figure 6. Histogram showing pre- and post-intervention responses on a zero to ten scale to the question: “Agrees with: Having HIV means I will eventually die from it.”

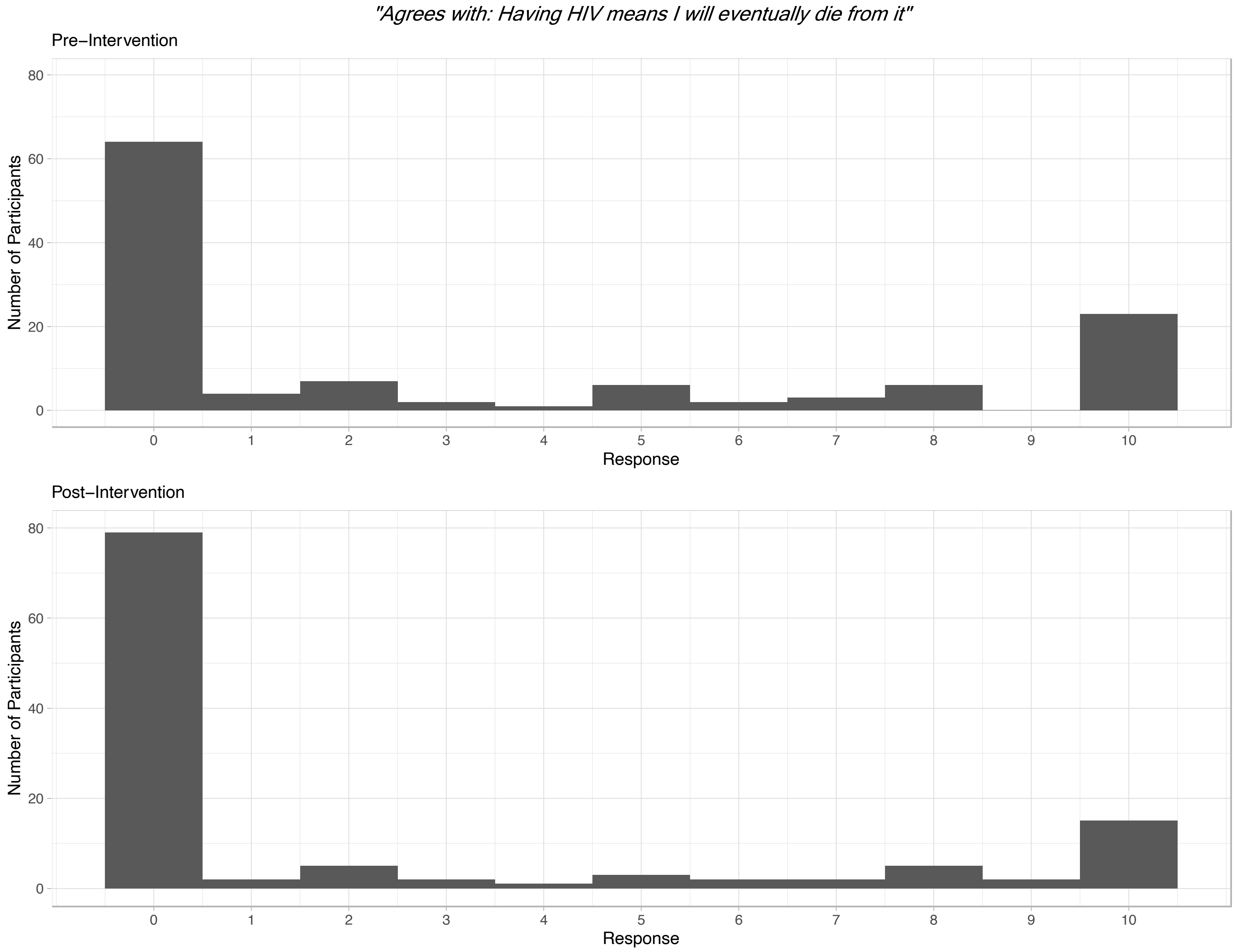

Supplementary Figure 7. Histogram showing pre- and post-intervention responses on a zero to ten scale to the question: “Imagine you were taking HIV treatment and had an undetectable viral load. How likely would you be to use a condom to prevent passing HIV to a sex partner who did not have HIV?”

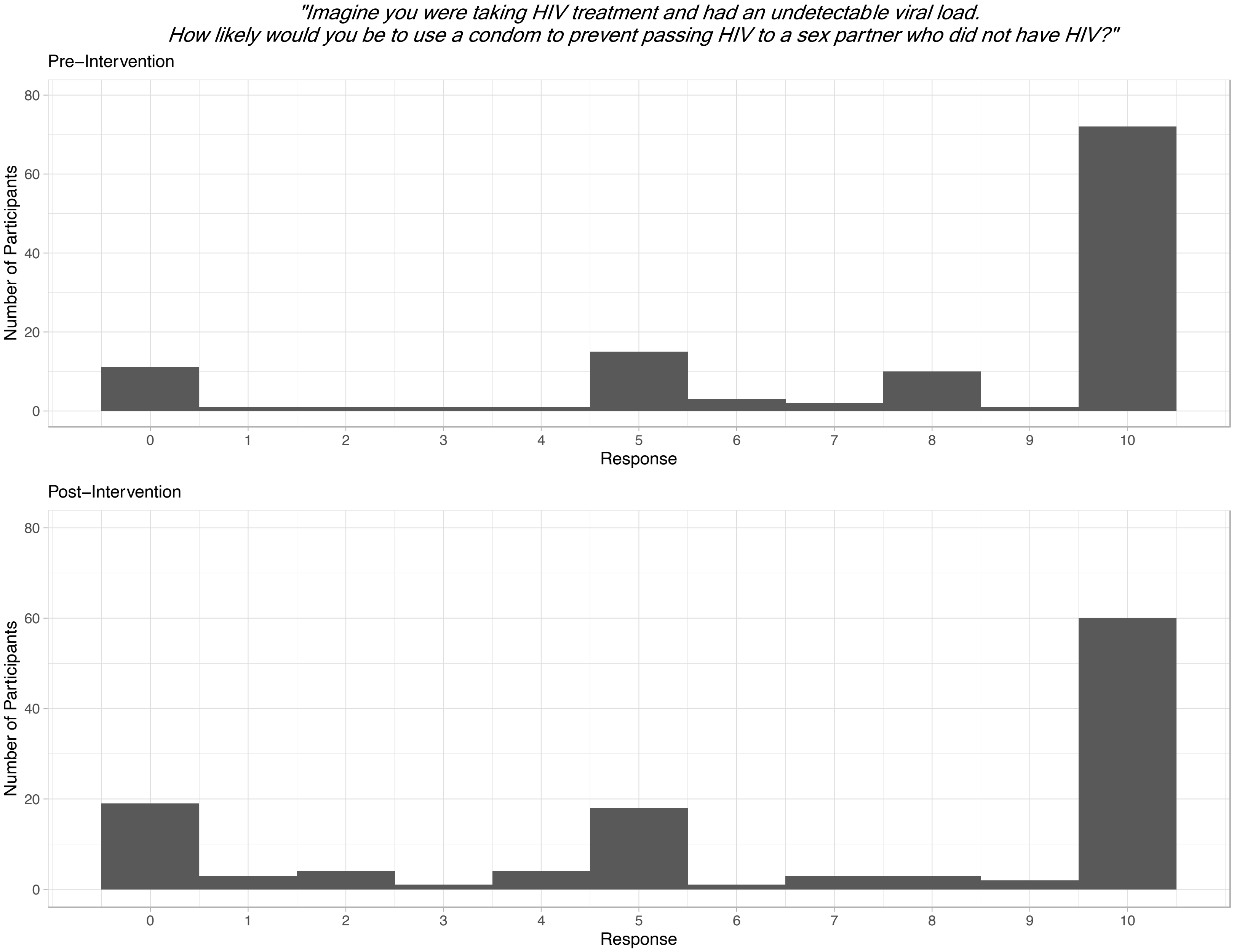

Supplementary Figure 8. Histogram showing pre- and post-intervention responses on a zero to ten scale to the question: “Agrees with: I feel motivated to stay on (or restart) HIV treatment.”

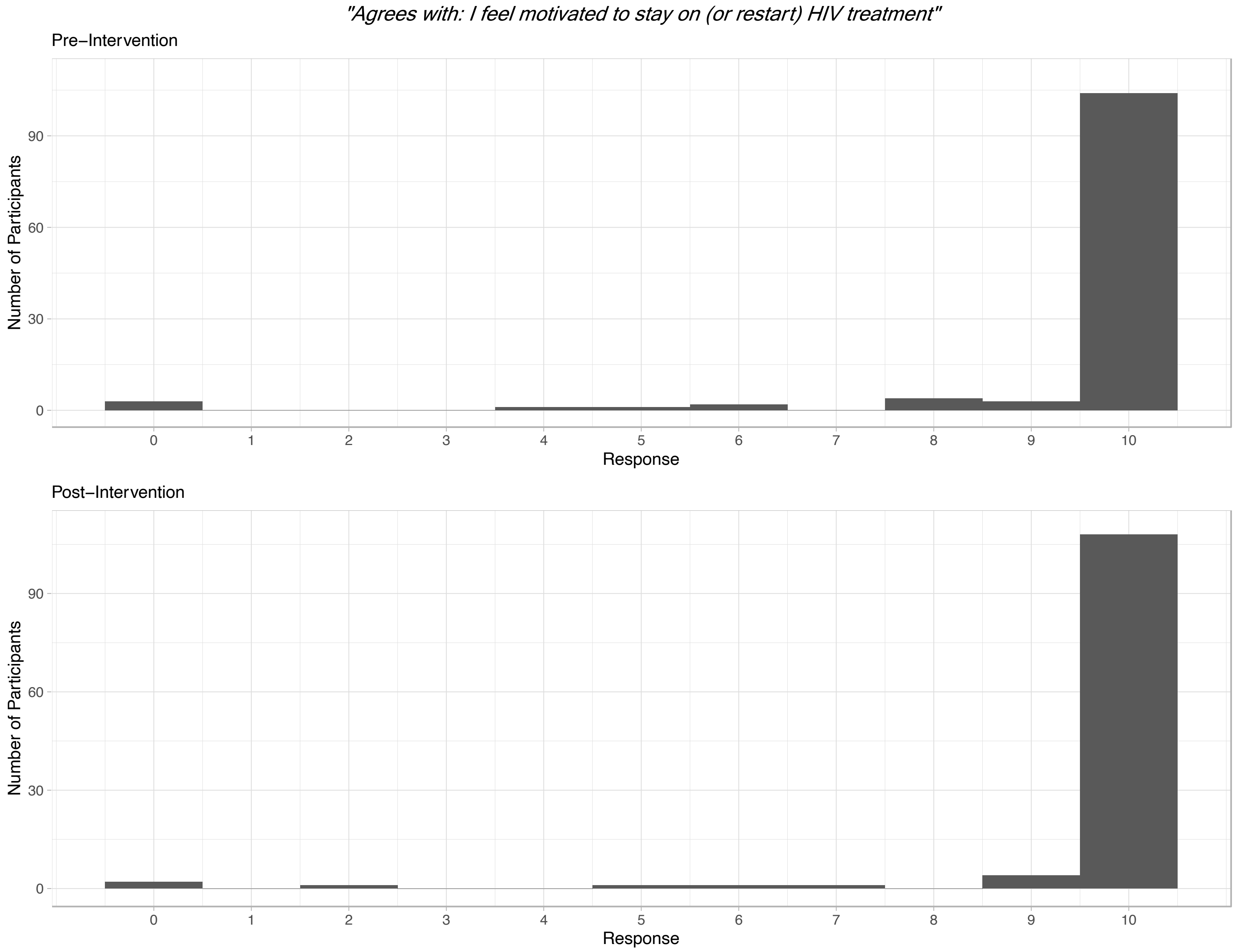

Supplementary Figure 9. Histogram showing pre- and post-intervention responses on a zero to ten scale to the question: "Agrees with: I understand my illness."

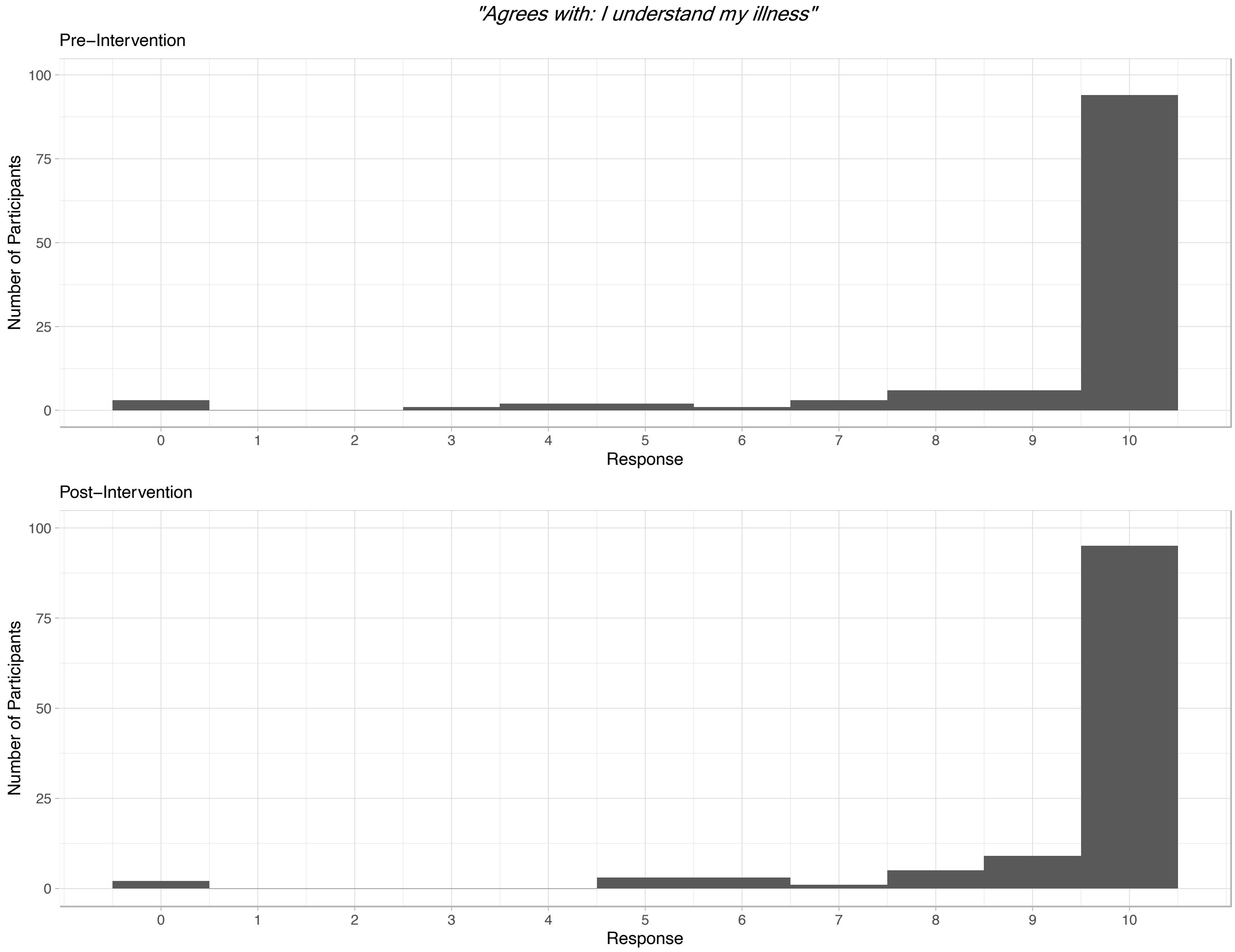

Supplement: Supplement 1 [file NIHPP2024.02.28.24303498v1-supplement-1.pdf]
